# Patient-Specific Surgical Implants Made of 3D Printed PEEK: Material, Technology, and Scope of Surgical Application

**DOI:** 10.1155/2018/4520636

**Published:** 2018-03-19

**Authors:** Philipp Honigmann, Neha Sharma, Brando Okolo, Uwe Popp, Bilal Msallem, Florian M. Thieringer

**Affiliations:** ^1^Hand Surgery, Kantonsspital Baselland, Liestal, Switzerland; ^2^Medical Additive Manufacturing Research Group, Department of Biomedical Engineering, University of Basel, Basel, Switzerland; ^3^Department of Oral and Cranio-Maxillofacial Surgery, University Hospital Basel, Basel, Switzerland; ^4^Apium Additive Technologies GmbH, Karlsruhe, Germany

## Abstract

Additive manufacturing (AM) is rapidly gaining acceptance in the healthcare sector. Three-dimensional (3D) virtual surgical planning, fabrication of anatomical models, and patient-specific implants (PSI) are well-established processes in the surgical fields. Polyetheretherketone (PEEK) has been used, mainly in the reconstructive surgeries as a reliable alternative to other alloplastic materials for the fabrication of PSI. Recently, it has become possible to fabricate PEEK PSI with Fused Filament Fabrication (FFF) technology. 3D printing of PEEK using FFF allows construction of almost any complex design geometry, which cannot be manufactured using other technologies. In this study, we fabricated various PEEK PSI by FFF 3D printer in an effort to check the feasibility of manufacturing PEEK with 3D printing. Based on these preliminary results, PEEK can be successfully used as an appropriate biomaterial to reconstruct the surgical defects in a “biomimetic” design.

## 1. Introduction

Reconstructive surgeries can be extremely challenging even to the most experienced surgeon especially due to complex anatomy, sensitivity of the involved systems, and uniqueness of each defect [1]. The need to reconstruct the defect in the best possible way along with time minimization for the surgical procedure is of crucial importance to surgeons for improving patient outcomes and well-being [2]. Patient-specific implant (PSI) can be an effective solution in this situation designed to fit precisely in the anatomical defects or malformations. The need to fabricate the PSI has led to many innovations and technological advancements in the field of medicine [3, 4].

The technologies, such as additive manufacturing (AM) also known as rapid prototyping (RP) or three-dimensional (3D) printing, are robustly growing and have positively influenced the biomedical sector over the last decade allowing the surgeons and researchers to utilize them in manufacturing objects [5, 6].

With its introduction in the late 1980s, along with a paradigm shift from the old mass production system of medical implants to customized implant production system, AM has attained a significant place in medical implant manufacturing industry [7]. Several organizations worldwide are manufacturing PSI using various AM technologies with computational tomography (CT) scan data [8]. Recently, the US Food and Drug Administration (FDA) increased their approval of 3D printed implants under the 510k (premarket notification) approval system. This will allow the healthcare providers to use the parts manufactured by AM in routine and for complex surgical procedures [2, 9].

AM works by building a model from the ground up, depositing the material in a layer-by-layer manner using digitally controlled and operated material laying tools [10]. AM is thus fundamentally different from traditional formative or subtractive manufacturing in that it is the closest to the “bottom up” manufacturing where we can build a structure into its designed shape using a “layer-by-layer” approach. This layer-by-layer manufacturing allows an unprecedented freedom in manufacturing complex, composite, and hybrid structures with precision and control that cannot be made through traditional manufacturing routes [11, 12].

Good initial image capture is imperative for creating accurate 3D printed models. The recent strides in imaging modalities have made it possible to create patient-specific anatomical models with greater precision. In addition, advances in segmentation software have made it increasingly easy to automatically or semiautomatically extract the surface of structures of interest from 3D medical imaging data [13, 14].

With all these advances, AM has emerged as a mainstream manufacturing technology in medicine for the fabrication of anatomical models, surgical implants, surgical guides, external aids, and biomanufacturing [15–25]. Various studies have been published suggesting the use of AM in 3D printing of cells, blood vessels, vascular networks, bones, ears, windpipes, and dental prosthetics including a jaw bone, and in future, even in corneas [26]. Surgeons can now fabricate 3D printed hand-holdable models (called biomodels) for the surgical task that can be used to educate the patient, plan the surgical approach, and act as an intraoperative surgical guide. These 3D printed medical models are being extensively used in orthopedic, cardiac, dental, and craniomaxillofacial surgeries with a potential to optimize patient treatment [27–31].

Currently, there are several technologies for AM like stereolithography (SLA), photopolymer jetting, selective laser sintering (SLS), electron beam melting (EBM), direct metal laser sintering (DMLS), and fused deposition modelling (FDM), which is also known as fused filament fabrication (FFF) [32, 33]. These technologies have emerged as a valuable tool for surgeons in reproducing anatomical objects as 3D physical models and are being used in the reconstruction of PSI [34].

Various alloplastic materials, such as metals, ceramics, polymers, and composites, are fabricated by AM technologies and are used in reconstructive and orthopedic surgeries. Due to their abundant availability, there are no concerns about the donor site morbidity, which is a huge disadvantage for autologous grafts [35].

Metallic implants including gold, tantalum, stainless steel, shape memory alloy, titanium alloy, and cobalt chromium alloy have been widely used in the hospitals either as permanent prostheses such as knee and hip prosthesis, cranial prosthesis, and dental implants or as temporary implants such as plates, pins, screws, and rods for the fixation of bone fractures. These implants have favorable mechanical strength and excellent friction-resistance and are the most preferred alloplastic material in AM for the manufacturing of orthopedic implants [36, 37]. However, their high strength and elastic modulus do not match to the normal human bone tissues and thus can cause a stress shielding effect leading to prosthetic loosening. In addition, the strong X-ray absorption of metals with respect to the surrounding tissues usually results in streak artifacts in the CT scan images. Further, as many metals are magnetic resonance imaging (MRI) incompatible, the possibility of examining the patient with MRI is limited. The long-term presence of metals* in vivo* can also trigger hypersensitivity reaction and initiate osteolysis [38]. These limitations also led to the exploration of ceramics as an alternative biomaterial.

Among ceramics, metallic oxides, calcium phosphate, and glass ceramics are commonly used. These materials exhibit favorable toxicity profile, good biocompatibility, and bioactivity. However, their low fracture toughness and ductility along with high modulus of elasticity and brittleness make them unacceptable for load-bearing applications [39].

Due to an array of limitations observed with metallic and ceramic biomaterials, more recently the use of polymers as a viable alternative is being explored. A large number of polymers, such as ultrahigh molecular weight polyethylene (UHMWPE), polymethyl methacrylate (PMMA), polylactide (PLA), polyglycolide (PGA), and polyhydroxybutyrate (PHB), are also widely used in various biomedical applications. However, only a limited number of polymers have been used for bone replacement purposes because they tend to be too flexible, and too weak for orthopedic and load-bearing implants applications [40].

Among the various alloplastic materials, polyetheretherketone (PEEK) has emerged as an attractive option for the PSI. PEEK is a semicrystalline linear polycyclic aromatic thermoplastic belonging to a family of linear aromatic polymers containing ether and ketone linkages [38].

PEEK was first developed by a group of English scientists in 1978 [41]. In the 1980s, PEEK was used as aircraft and turbine blades and, by the late 1990s, PEEK was used to replace metal implant components, especially in orthopedic and trauma specialities. PEEK has since been used in a wide range of applications owing to its excellent combination of high-temperature performance, chemical resistance, fatigue resistance, lightweight, high yield strength, stiffness, and durability [38].

Although manufacturing and 3D printing of PEEK polymer have been widely investigated in different industries, its use in the medical field is challenging due to its physical properties [42, 43].

In this article, we present the preliminary results and technical aspects on the material extrusion (FFF, 3D printing) based fabrication process of PEEK parts with a focus on PSI for surgical applications.

## 2. Materials and Methods

### 2.1. PEEK Filament

**Figure 1:**
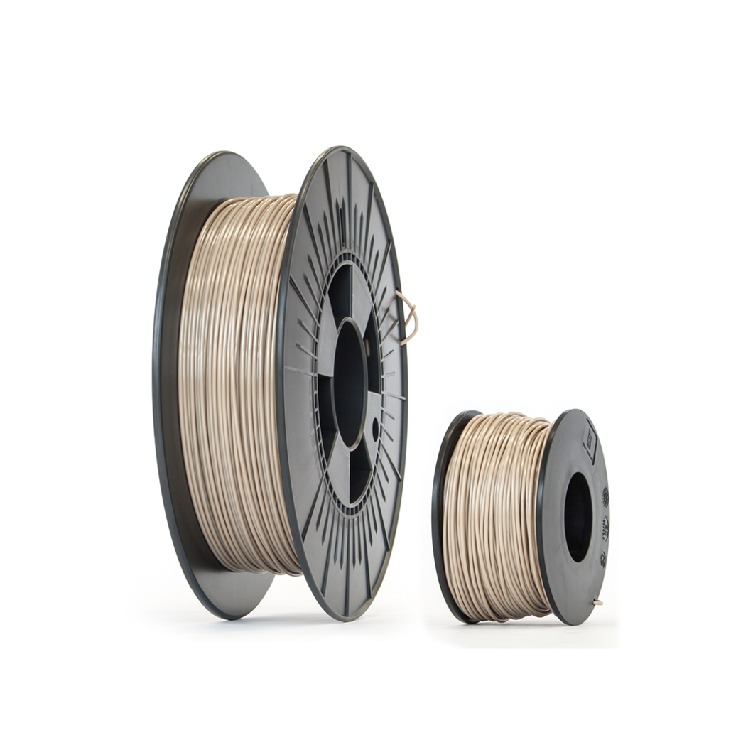
Medical grade PEEK filament. https://apiumtec.com/de/new-peek-printing.

For the printing process, Apium PEEK 450 Natural 1.75 mm filament produced from medical grade PEEK granules was used (Supplier: Apium Additive Technologies GmbH, Karlsruhe, Germany; Manufacturer: Evonik Industries AG, Germany). This filament is a semicrystalline polymer with density of 1.30 g/cm^3^ and tensile strength of 97 MPa (Figure 1). With excellent chemical resistance, it is a perfect combination of strength, toughness, and stiffness. Additionally, it is very tolerant to gamma radiation, is extremely stable against hydrolysis, and is suitable for sterilization.

### 2.2. PEEK FFF 3D Printer

**Figure 2:**
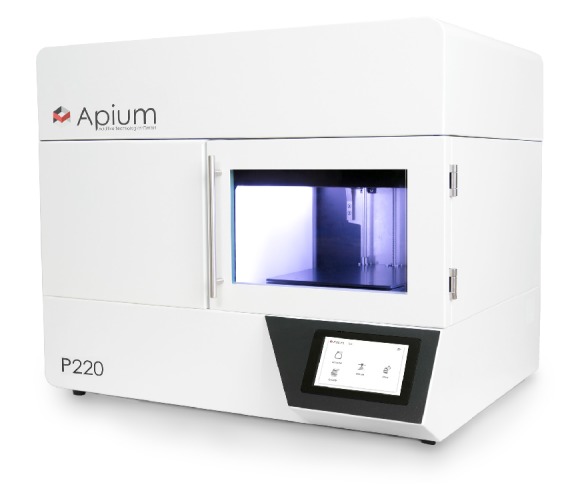
PEEK FFF 3D printer (Model P220).

The FFF 3D printer used in our study was a prototype of the Apium P220 (Figure 2), based on the FFF technology (Apium Additive Technologies GmbH, Karlsruhe, Germany). The printer uses Apium Controlling Software (ACS) with 65 adjustable parameters utilizing Standard Tessellation Language (STL) format files.

The performance and technical specifications of the FFF 3D printer are mentioned in Tables 1 and 2.

### 2.3. FFF 3D Printing Process

FFF starts with a 3D computer-aided design (CAD) model of the implant, exported as an STL file from a CAD modelling software program. The STL file is sliced by the computer slicing software into horizontal layers that are as high as the layers in the 3D printer machine. A rod-shaped filament is supplied to the machine through a feeding tube. The molten thermoplastic material is extruded through one nozzle (diameter 0.4 mm, computer controlled) and deposited layer-by-layer following a specific laydown pattern. The nozzle follows a raster pattern in the *X*, *Y* plane and forms a layer. Later, a layer deposition is finished, the working bed in the *Z* direction is lowered, and the new layer is extruded. With complex anatomical geometries, support structures are incorporated and the 3D object including support structures is printed layer-by-layer fusing the layers together. A special fixative (DimaFix, DIMA 3D, Valladolid, Spain) spray was applied to the “cold” print bed for adhesion before printing. The entire chamber was enclosed so that recommended bed temperature of about 100°C and print temperature of about 400°C can be achieved.

### 2.4. Digital Data Acquisition and Preparation

**Figure 3:**
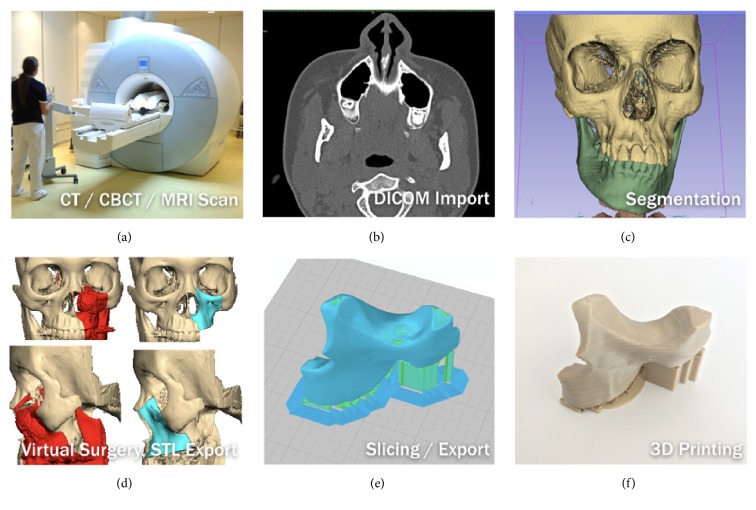
Workflow to generate a 3D model.* Thieringer FM*.

For the anatomical data modelling, the representative models of the patient's anatomical data were constructed based on radiological raw data of the patient obtained in a Digital Imaging and Communications in Medicine (DICOM) format from CT scan data. In DICOM format, the data was presented in a series of slices through the patient's anatomy, with slice thickness between 0.3 and 0.6 mm depending on the anatomical region. A medical modelling software program (Mimics; Materialise, Leuven, Belgium) was used to compile the DICOM data into axial, sagittal, and coronal planes. Following this, threshold selection was done, in which the inbuilt greyscales for bone are selected to mark a particular anatomical tissue type. Using segmentation, a virtual 3D model of the anatomical region was thus created. The 3D virtual model created in Mimics was exported to 3-Matic (Materialise, Leuven, Belgium) for further processing, design, and construction of PSI. The final data sets were converted and exported as an STL file and sent to the 3D printer, which finally fabricated the PSI by FFF. The overall sequential process is displayed in Figure 3.

## 3. Results and Discussion

### 3.1. Results

During this preliminary evaluation, five different PEEK structures were fabricated as follows.

**Figure 4:**
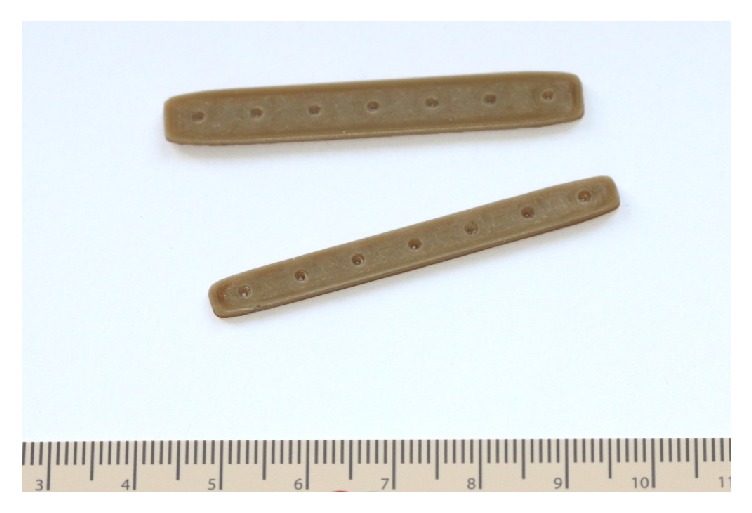
3D printed PEEK osteosynthesis plates.* Thieringer FM et al. AMPA 2017*.

**Figure 5:**
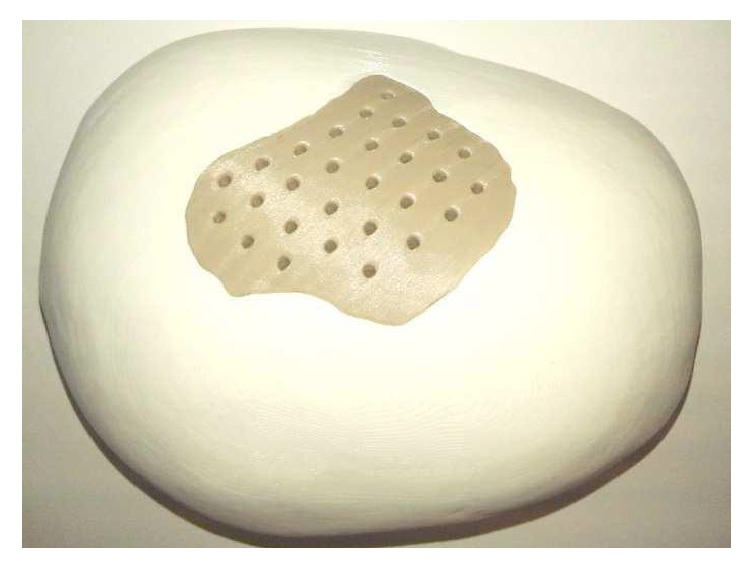
3D printed PEEK cranioplasty PSI for repair of defects in the cranial vault.* Thieringer FM et al. AMPA 2017*.

**Figure 6:**
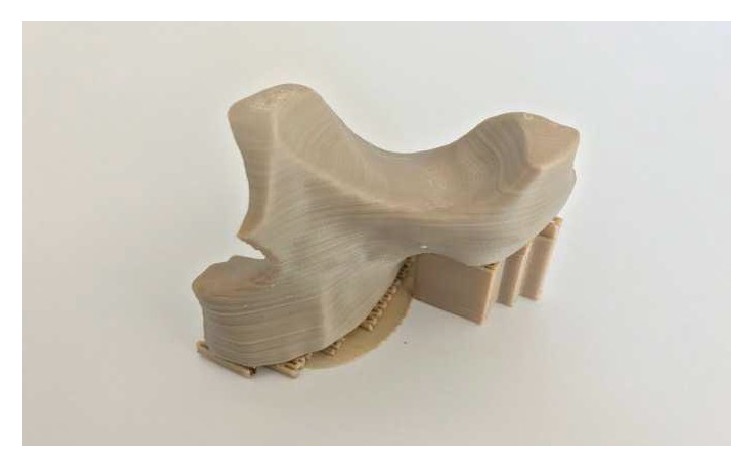
Lightweight midface-zygomatic bone PSI with support structures for immediate replacement.* Thieringer FM et al. AMPA 2017*.

**Figure 7:**
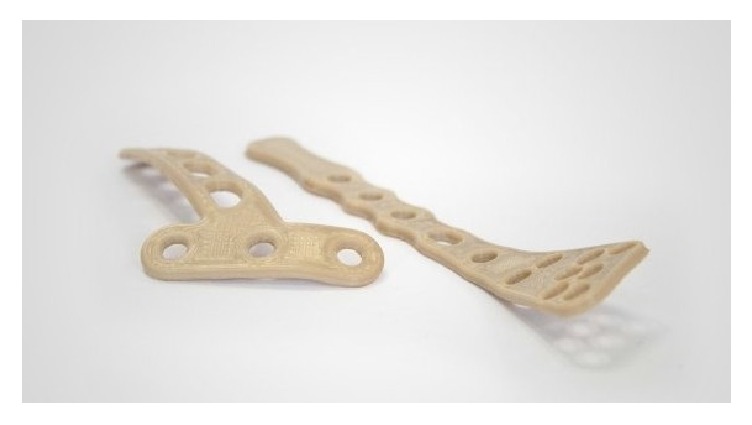
PSI small fragment osteosynthesis plates.* With permission of Apium GmbH*.

**Figure 8:**
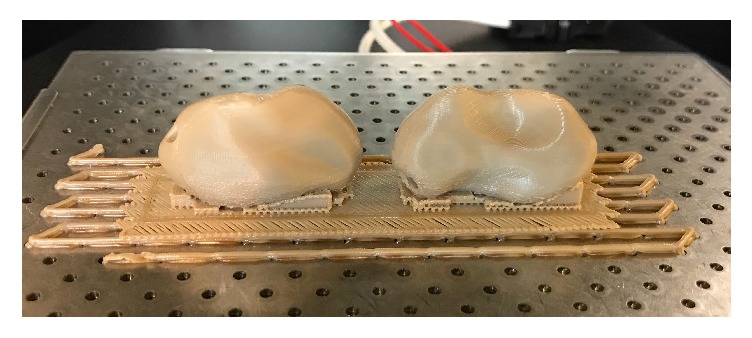
Prosthetic implant for scaphoid bone replacement (green body), patent pending (EP15195745.1 PCT).

- Osteosynthesis plate (Figure 4)
- Cranioplasty PSI for repair of defects in the cranial vault (Figure 5)
- Lightweight midface-zygomatic bone PSI with support structures for immediate replacement (Figure 6)
- Small fragment PSI osteosynthesis plates (Figure 7)
- Prosthetic implant for scaphoid bone replacement (Figure 8)

The fabrication results showed that the 3D printed PEEK PSI were of a smooth finish without any irregularities. No black-specks formation nor discoloration (improper crystallization) was detected in the test parts. All of the 3D printed parts passed a certified sterilization test without any deformation. Thus, these preliminary tests confirm the possibility of fabricating 3D printed PEEK in the desired way (extrusion through nozzle) by FFF.

## 4. Discussion

Over the past few years, PEEK has attracted a great deal of interest from material scientists and orthopedists. It is suitable for load-bearing implants because of its favorable biomechanical properties, radiolucency, MRI compatibility, and chemical inertness [44, 45]. PEEK has primarily been used in spine surgery for interbody fusion cages. PEEK has also been used in combination with other materials such as reinforced carbon fiber (CF/PEEK), for fracture fixation and prosthesis (e.g., artificial hip joints) [46–49]. Various studies conducted with PEEK in reconstruction of complex maxillofacial defects and calvarial defects have also shown excellent postoperative esthetic and functional results without any complications [41, 50–52]. Hence, PEEK is a suitable biomaterial and an appropriate alloplastic material for reconstructive and orthopedic surgeries.

Until now, PEEK medical implants can only be manufactured by traditional subtractive manufacturing methods, with the use of Computer Numerical Controlled (CNC) machine. This technique usually starts with a blank block of PEEK material that is slowly shaped into the final part. The computer controls the tools needed for fabrication of the part by controlling the lathes, mills routers, and grinders used in the process. Furthermore, additional postprocessing work needs to be done after fabrication. This technique is time consuming resulting in substantial waste generation and is far more expensive than AM [53]. Additionally, as mentioned earlier the use of PEEK polymer in 3D printing is challenging due to its physical properties [42, 43].

Technological advances have recently provided techniques such as 3D printing of PEEK using FFF, which can create various CAD forms. FFF, being a low-cost technique with a short start-up time, provides a major advantage over other manufacturing techniques. In this technique, the PEEK polymer material in the solid state is thermally brought to a flow regime and then solidified through a thermal gradient. As rheology and heat transfer characteristics are two important properties of FFF thermoplastic materials, the interplay between cooling rate and material flow behavior needs to be fully moderated in this technique in order to create parts with an appreciable high dimensional accuracy [54].

With the introduction of CAD and computer-aided manufacturing (CAM) techniques in surgery, it is now possible to fabricate implants in various forms and designs with biocompatible materials. The 3D printed PSI are used in a wide range of applications in the medical field. Our research focused on the surgical fields in which PEEK is already being used and fabricated either by milling or by injection molding techniques. However, as these manufacturing techniques are expensive and material-consuming, with the introduction of FFF 3D PEEK printers, fabrication of PSI is conceivable, providing substantial benefits to the surgical fields.

Osseous integration of PEEK depends on the surface composition, surface energy, surface roughness, and topography [55]. With the standard production techniques, the surface structure of PEEK is inert and smooth [38]. However, with FFF 3D printing, PEEK surface properties can be modified and fabricated to yield either rough or smooth surfaces.

Osteosynthesis materials made out of PEEK are already being used in hand and trauma surgeries especially for treating distal radius fractures. With FFF, 3D printed PEEK patient-specific plates (Figure 7) can be produced in a short period at a very low cost. This plays an important role in general trauma and orthopedic surgeries as 3D printed PEEK PSI can be readily available for use within 24 hours after admission in the hospital. As a proof of concept, we also test printed a standard osteosynthesis plate (Figure 4) and a scaphoid bone replacement prosthesis (Figure 8).

Until now, the reconstructive surgeries for congenital and acquired defects of the skull and facial regions are reconstructed with standard manufactured PEEK implants [56]. However, with FFF, 3D printing of these PSI is now possible (Figures 5 and 6) and defects can be easily treated in a short period of time with this low-cost and in-house printing facility at the hospitals [57]. Our results, thus, suggest that FFF has the ability to manufacture complex implant structures with unlimited geometries that could not have been possible with traditional milling techniques.

Conversely, manufacturing PEEK by FFF itself is quite complex and various parameters interactions have to be considered. Formation of black-specks can potentially develop in the printed parts or at the regions of the printer where the melt exits, such as the nozzle as well as areas around the nozzle. These deformations suggest uncontrollable thermodynamically driven changes within the melt. Possible sources of black-specks in FFF 3D printed PEEK are (1) degradation of the molten filament at the joint of the heat-break and nozzle, (2) degradation of the melted filament inside the nozzle shaft, (3) poorly designed nozzle tip-area such that the melt collects at the exposed surface and then degrades, (4) irregular thermal loading of the melt by the heating elements, (5) melt degradation due to presence of foreign particles interfacing with the melt, (6) prolonged residency of a melt-batch in the nozzle shaft/barrel, and (7) too high processing temperature. Therefore, one of the critical factors for 3D printing of PEEK by FFF is continuous maintenance of high temperature for material extrusion.

With the introduction of an all metal hot-end extruder in the printer used in our study, it is possible to attain uniform temperatures up to 540°C, and the enclosed chamber provides an efficient heat management for continuous printing. The bed temperature and the print temperature of the printer are maintained high enough to provide a good thermal control over the entire build chamber leading to good layer bonding and thereby prevents “specking” in PEEK parts. This was evident from the various structures created during the present study where such black-specks were not observed [53].

The preliminary findings from our study suggest that anatomically complex PSI can be printed using an FFF 3D printer. The authors strongly believe that FFF has a huge potential and can provide various advantages such as less wastage of material, cost-effectiveness, low investment on machine, easy operator training, faster in-house implant production, and a better personalized patient care approach. All these factors have a potential effect in reducing the financial burden on the overall healthcare sector.

### 4.1. Study Limitation

Along with a requirement of support structures in complex geometries, another important aspect that needs to be addressed is the effect of anisotropy on FFF 3D printed parts. In FFF, a mechanical adhesion (not chemical) is created within the layers of the polymer and, thus, the printed objects have different mechanical properties based on the direction of mechanical stress applied on them. This means that along a particular line deposition pattern, the part will be stronger in the direction of the deposited line and relatively less strong along the axes that are primarily composed of interfiber bonding regions, namely, the two spatial axes orthogonal to the line axis.

As in many spinal and craniomaxillofacial applications, the mechanical stresses are essentially directed along a specific axis and an anisotropic response from the implant can be advantageous, and future experiments to address this behavior are needed. Further, the part testing needs to be done according to International Organization for Standardization (ISO)/American Society for Testing and Materials (ASTM) standards to make FFF 3D printed PEEK usability beyond PSI.

## 5. Conclusion

Personalized medicine is poised to revolutionize the modern practice of medicine where “one size does not fit all” and implants must be tailored to individual patient's needs, which are the ultimate goal. The refinement of imaging technologies, coupled with the capabilities to fabricate PSI, has given rise to a proliferation of alternatives to traditional off-the-shelf implants. With the availability of inexpensive compact desktop 3D printers, the surgeons in near future can manufacture medically certified 3D PSI in their own hospitals. This would have a major advantage for surgical planning, thereby reducing an enormous amount of time compared with the off-site implant production by third-party providers leading to a more cost-effective healthcare management. Although few regulations specifically targeting AM for medical devices currently exist, regulation by the FDA and other bodies is expected to increase in the coming years making the approval and manufacturing of new device classes at companies or at hospitals a lengthy process.

From the requirement of clinical trial data, pre- and postmarketing approvals, vigilance reporting timelines, data transparency, and unique device identification (UDI), to name a few, various regulatory measures will be needed to be adhered to, so as to make the medical device available to the patients.

Though this article presents only a small amount of the research done in the project to fabricate 3D printed PEEK PSI using FFF, it indeed opens up a huge scope for innovation and future development in the surgical applications.

## 6. Further Steps

Future development is planned to improve the mechanical properties, so some more tests with appropriate or additional knowledge on part orientation and equipment parameters will be done.

**Figure 9:**
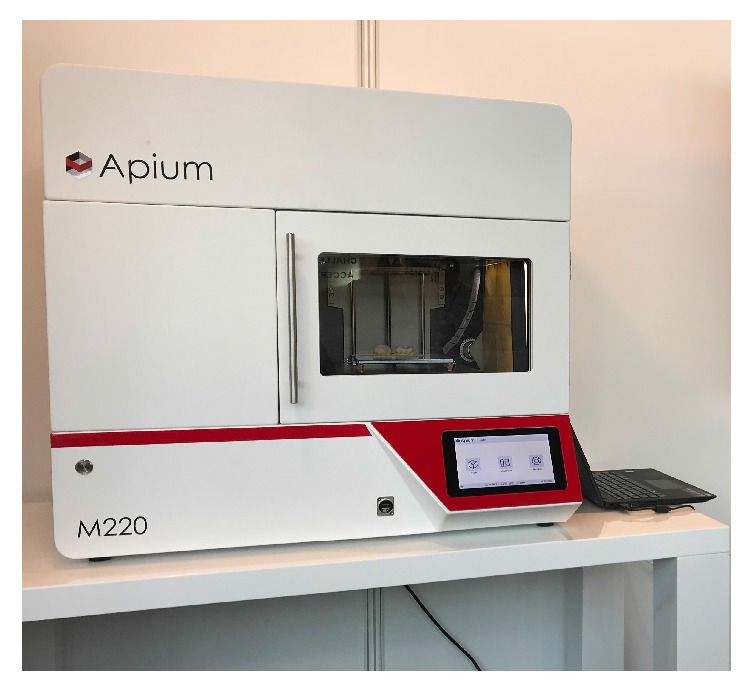
Medical version of the PEEK FFF printer (Model M220).

Within the framework of the cooperation of the institutions listed above, a medical version based on the P220 of this PEEK FFF printer (Figure 9), which has been introduced to the industrial market for some time, is currently undergoing the certification process for medical applications. The test specimens required for the certification were prepared, evaluated, and passed through other test methods (e.g., cleaning, sterilization).

Additionally, the integration of 3D printing is additionally examined from the medico-legal point of view in the clinical environment.

## Figures and Tables

**Table 1:** Performance specifications of the FFF 3D printer.

Parameter	Performance specifications
Print bed volume (*w*, *d*, *h*)	155 × 155 × 155 mm
Print volume (*w*, *d*, *h*)	140 × 135 × 148 mm
*x*/*y* resolutions	Product resolution: 0.5 mm, machine resolution: 0.0125 mm
*z* resolution	Product resolution: 0.1 mm, machine resolution: 0.05 mm
Reproducibility	0.1 mm
Minimum layer thickness	0.1 mm
Maximum layer thickness	0.3 mm

**Table 2:** Technical specifications of the FFF 3D printer.

Parameter	Technical specifications
Number of extruders	1
Nozzle diameter	0.4 mm
Filament diameter	1.75 mm
Print head temperature	Up to 520°C
Print bed temperature	Up to 160°C
Size (*w*, *d*, *h*)	590 × 620 × 680 mm
Slicing software compatible	slic3r and Simplify3D
